# Identifying barriers and strategies for achieving competency in removable prosthodontics in undergraduate dental students: a mixed-method study

**DOI:** 10.1186/s12903-024-03874-x

**Published:** 2024-01-23

**Authors:** Watcharapong Mongkolrattanasit, Veerit Tanvarasethee, Kittapob Thangjantaraprapab, Supachai Chuenjitwongsa, Nareudee Limpuangthip

**Affiliations:** 1 Faculty of Dentistry, Chulalongkorn University, Bangkok, Thailand; 2 Department of Biochemistry, Faculty of Dentistry, Chulalongkorn University, Bangkok, Thailand; 3 Department of Prosthodontics, Faculty of Dentistry, Chulalongkorn University, Bangkok, Thailand

**Keywords:** Dental education, Competency, Prosthodontics, Tacit knowledge, Threshold concept

## Abstract

**Background:**

Developing competency in removable prosthodontics (RP) is challenging for undergraduate dental students because it involves threshold concepts and tacit knowledge. Understanding this process can enhance learning and professional development in RP. The objective of this study was to identify the barriers hindering knowledge (threshold concept) and skill (tacit knowledge) development, and to propose strategies for achieving RP competency.

**Methods:**

Adopting critical theory, quantitative and qualitative approaches were implemented. The participants were third- to sixth-year dental students and recent dental school graduates. An online questionnaire was used to investigate the knowledge and skills required for achieving RP competency and barriers to RP competency development. Four focus groups were conducted to gather in-depth information. The data was analyzed using descriptive statistics and thematic analysis.

**Results:**

A total of 322 respondents completed the questionnaire (67% response rate), and 26 of them participated in focus group interviews. The four threshold concepts to achieve RP competency were the basic principles of RP, removable partial denture design, occlusion, and dental materials. The two main tacit knowledges were impression making and material handling skills. The curriculum should integrate strategies to assist dental students in overcoming intrinsic barriers such as self-experience, revision, and spatial-temporal relationship, along with extrinsic factors such as clinical correlations of content, discussions, and immediate feedback.

**Conclusions:**

Threshold concepts and tacit knowledge in RP for undergraduate dental students have been proposed The strategies to overcome barriers comprise intrinsic and extrinsic factors that include the adoption of experiential learning. This study suggests effective teaching methods and learning strategies to maximize student learning and RP competency development when designing the undergraduate RP curriculum in dental education.

**Supplementary Information:**

The online version contains supplementary material available at 10.1186/s12903-024-03874-x.

## Background

Removable dental prostheses, including partial and complete dentures, are widely used to replace missing teeth in edentulous individuals to restore oral function and quality of life [1]. Dental students must achieve core competencies during the undergraduate curriculum to provide removable denture services according to professional standards. Competency in dental education integrates knowledge, skills, attitudes, and character for safe, ethical, and independent practice [2, 3]. Competency is a key stage in the development of expertise, known as the novice-expert continuum, encompassing the novice, beginner, competent, proficient, and expert stages [2, 4]. Beginner is the stage when learners gradually enhance their decision making-skills and apply knowledge and skills in different contexts [5]. To instill confidence and competence in students, it is essential to address the threshold concepts, which are barriers to knowledge development, and tacit knowledge, which is the barrier to skill development, especially in removable prosthetic dentistry.

Threshold concepts are core concepts within a field that act as bottle necks or barriers to overcome before achieving mastery in that particular field [6, 7]. Threshold concepts include but not limited to, troublesome (difficult to learn), integrative (related to other subjects), liminal (a fluctuation between old and new conceptual understanding), transformative (a new way of thinking), and irreversible (difficult to be unlearned) [7–11]. Once individuals develop deep learning on these concepts, they can apply knowledge, which is unlikely to be forgotten, in other contexts. Meanwhile, tacit knowledge refers to knowledge that is challenging or impossible to transfer from instructors to learners or from one individual to another. Such knowledge occurs in a non-cognitive part of the brain; thus, it is commonly learned through observation and practice [12, 13]. In dentistry, tacit knowledge involves cognitive ability and psychomotor skills, requiring linkages between both aspects for effective learning [13]. Removable prosthodontics (RP) requires an understanding of fundamental concepts, skills, and clinical experience. This extends beyond dental prosthesis fabrication to include an effective patient management. Identifying threshold concepts and tacit knowledge in RPs poses challenges, particularly for novice and beginners with lacking prior clinical experiences as they progress towards competence.

The undergraduate dental education system in Thailand follows a 6-year program, comprising 3 years of preclinical study and 3 years of clinical practice. Lectures and laboratory practice related to removable prosthodontics begin in the 3rd and 4th years, while clinical practice takes place during the 5th and 6th years. According to the Competency Standards for Thai General Dentists, dental prosthodontic competency for undergraduate students encompasses the fabrication of temporary and permanent dental prostheses in non-complex cases, including fixed and removable dentures [14]. This competence also involves providing oral hygiene instructions to patients with dental prostheses, effective communication between dentists and dental technicians, evaluating the quality of dental prostheses, and making necessary adjustment. In addition, competent undergraduate dental students must be capable of explaining the indications, contraindications, and overall process of implant-retained dental prostheses [14]. It is noted that non-complex cases exclude implant-retained prostheses, full-mouth rehabilitation which involve significant alters vertical dimension or maxillo-mandibular relationships, as well as dental prosthesis for restoring maxillofacial defects. Meanwhile, complex cases are generally reserved for postgraduate dental students who are seeking to attain a higher level of proficiency. In addition to managing complex situations, proficient individuals, should have the ability to perceive situations holistically, rather than as separate components, and to prioritize elements correctly in a sequential manner [2, 3].

Several factors can affect the capability of dental students to achieve RP competency, including learners, instructors, and learning environment [15–17]. To overcome these barriers, experiential learning is utilized to develop learning through practice [18]. Experiential learning, which is generally used by health professionals, involves four stages: concrete experience (engage in tasks or situations), reflective observation (reflect on the experience), abstract conceptualization (create personal learning concepts), and active experimentation (apply the concepts in new scenarios) [19]. This cycle leads to meaningful learning from experience; however, the stages may not follow a strict sequence for effective learning [18, 19].

In the field of RP, undergraduate students often face challenges in applying their knowledge from didactic and laboratories to clinical practice, especially in this study context. To our knowledge, no report exists on the threshold concept and tacit knowledge in RP. Thus, the objectives of this study were to identify the barriers hindering knowledge (threshold concept) and skill (tacit knowledge) development, and to propose strategies for achieving RP competency in the undergraduate curriculum.

## Methods

### Study design and ethical approval

This study adopted a critical theory paradigm, employing an explanatory mixed-method (i.e., using quantitative method for gathering initial trends and using qualitative method for explaining quantitative data). Within the critical theory paradigm, the present study aimed to explore the transformation of the reality (teaching and learning RP) that has been shaped over time and influenced by environmental factors, indicating a qualitative study in nature. The quantitative aspect was employed solely to complement the qualitative method, and thus, no hypothesis was formulated. The participants included third- to sixth-year dental students in the undergraduate program at the Faculty of Dentistry, Chulalongkorn University in the academic year of 2022, and recent dental school graduates with the eligible criteria as follows.

Inclusion criteria:

- Pre-clinical students (third and fourth-year students) who were taking or completed removable complete denture and partial denture lectures and laboratory courses.
- Clinical students (fifth and sixth-year students) who had begun removable prosthodontic clinical practice.
- Recent graduates who had graduated within the past two years (academic year of 2020 and 2021).

Exclusion criteria: Those who declined to participate in the questionnaire.

Quantitative data were acquired through an online survey, chosen for its efficiency in gathering data from a large sample group. For qualitative insights, two primary approaches were considered: (1) individual interviews for in-depth information with maintained confidentially, and (2) focus group interviews for dynamic group discussions providing more insight from diverse perspectives [20]. Ultimately, focus group interviews were selected to encourage group interaction and discussion, aiming to obtain more comprehensive results.

Data was collected from December 2022 to February 2023, during the prosthodontic courses. The students were informed about the study’s objectives and procedures. The study was approved by the Human Research Ethics Committee of the Faculty of Dentistry (study code: HREC-DCU 2022-115).

### Data collection

The study comprised two parts: quantitative online questionnaires and focus group interviews. The quantitative online questionnaires were constructed by documentary analysis. Information from the course syllabi of RP courses were collected, including related subjects, minimum competency standards for Thai dentists, prosthodontic textbooks, and course evaluations. The data were analyzed to create a framework of the knowledge, skills, and barriers to achieve RP competency. The findings were used to formulate questions for the survey questionnaire and focus group interviews.

The questionnaires for quantitative data collection were validated by 6 experts in the prosthodontic department and a pilot study was performed using 30 participants from the same sample group. An online questionnaire was distributed using Google form to 484 members of the target population, comprising 198 pre-clinical, 193 clinical dental students, and 93 recent dental school graduates. Two sets of questionnaires were developed for the preclinical students, and the clinical students plus recent graduates (Supplementary file). Each questionnaire covered 3 parts: Part I gathered demographic information, Part II contained main questions to identify the key knowledge and skills required for achieving RP competency and their barriers. A four-level ordinal scale was used to rate the individuals’ perceptions of essentiality and difficulty for each topic. For the pre-clinical students, this part solely focused on the difficult topics because they had been exposed to only didactic and laboratory courses. Part III included open-ended questions for detailed comments on the reasons. The investigators and 4 student representatives distributed the Google form link, with reminders sent 2 weeks later. The acceptable response rate of a survey questionnaire was set at least 70% [6]. The survey questionnaire data was analyzed using descriptive statistics with IBM SPSS statistical software version 29.0.

For qualitative data collection, the focus group questions were developed based on documentary analysis and were triangulated with the quantitative data obtained from the online questionnaires. The interview questions were validated by the same experts who validated the online questionnaire. Four focus-group sessions were conducted using a semi-structured interview. The participants comprised 7 preclinical, 9 clinical dental students, and 7 recent dental school graduates, selected through convenient and purposive sampling. They were asked a list of predefined questions about the key factors related to barriers and solutions in learning and developing competency in RP. Data collection continued until no new information was presented by the participants. The voice records were transcribed, deconstructed, and coded for thematic analysis. Regular referring to the original transcript and quotations was maintained during data analysis, ensuring the validation of data interpretation, and promoting result transparency. Two investigators (W.M. and V.T.), trained in thematic analysis, independently analyzed the transcription of the focus group sessions. The analyzed data were compared, and any discrepancies were discussed with 2 other investigators who were experts in dental education (S.C.) and prosthodontics (N.L.) to reach a consensus. The criteria for threshold concept and tacit knowledge were adopted from the literature [10, 13].

All procedures were conducted in Thai language. The questionnaire, quotations, and results were translated into English and cross-verified among three dental student investigators (W.M, V.T., and K.T.). The validation of the translation was performed through triangulation between the dental students and confirmed by one prosthodontist (N.L.), one specialist in dental education (S.C.), as well as two experts whom English is their first language (one dentist and one engineer).

## Results

The quantitative online questionnaires underwent validation, with unanimous approval from experts regarding the questionnaire’s content. In the pilot study, all 30 students successfully completed the questionnaires without major comments. Therefore, only minor adjustment for typos and writing styles were made, and no additional statistical analysis was employed.

**Fig. 1:**
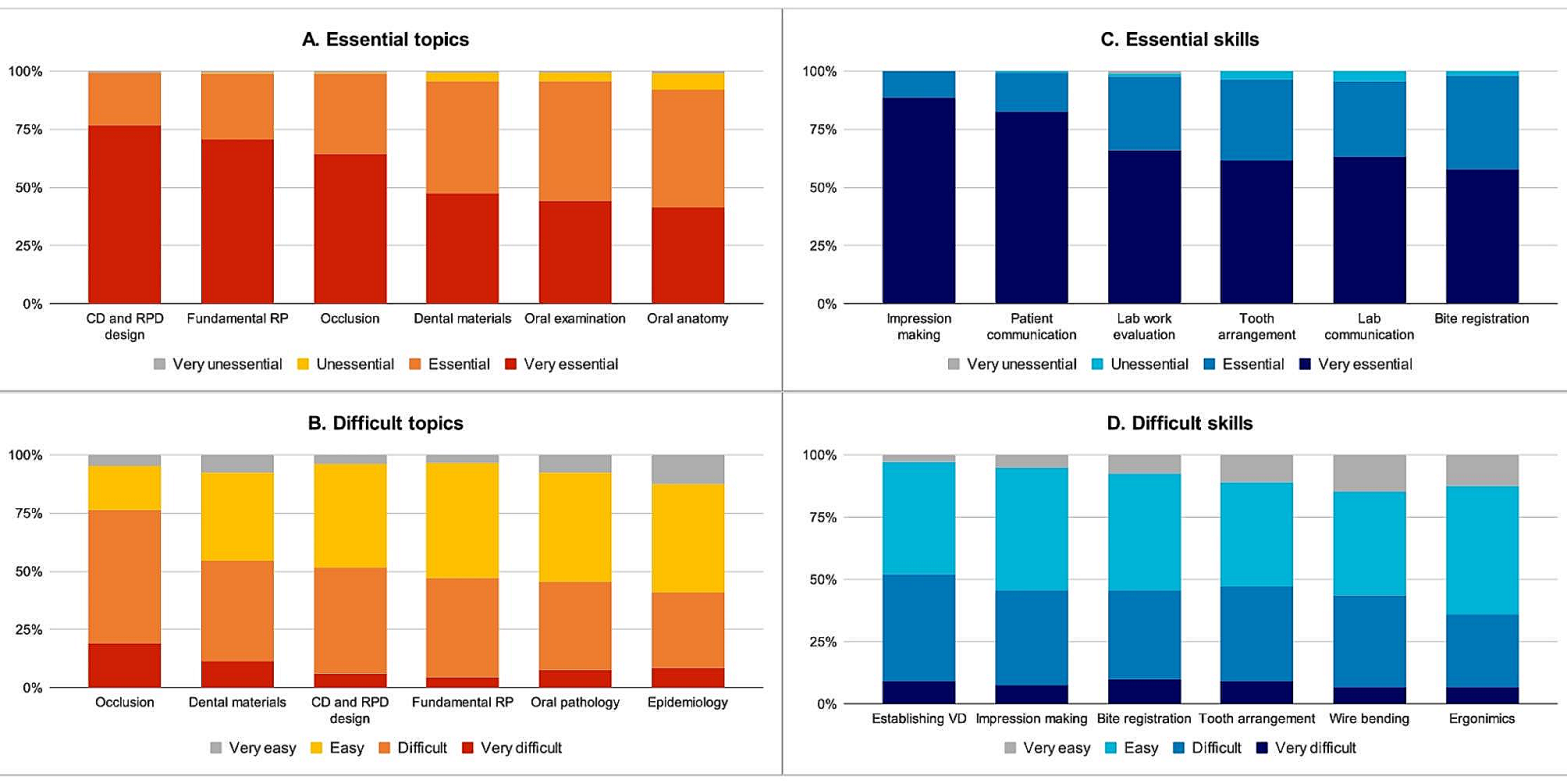
Perception on the essentiality and difficulty of topics and skills related to removable prosthodontic learning and practicing. A, Essential topics (*N* = 176). B, Difficult topics (*N* = 322). C, Essential skills (*N* = 176). D, Difficult skills (*N* = 176)

**Fig. 2:**
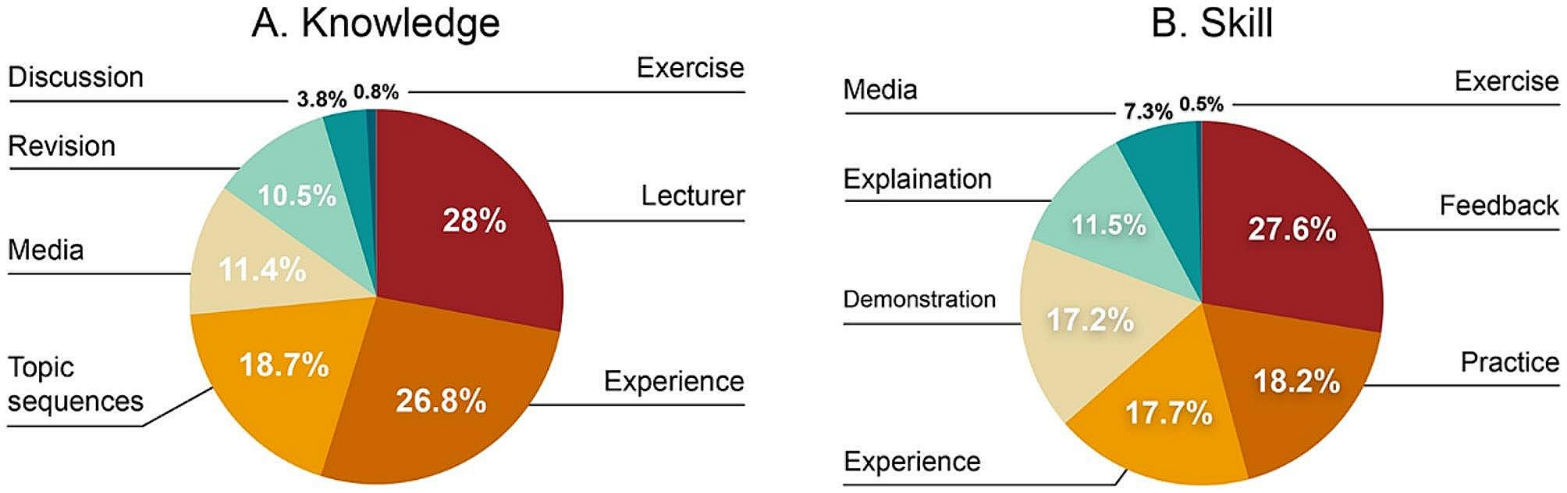
Factors related to knowledge and skill development. **A**, Knowledge; **B**, Skill

A total of 322 participants completed the online questionnaire comprising 146 pre-clinical students, 131 clinical students, and 45 recent graduates. The overall response rate was 64%. The participants had a mean age of 23.3 (± 2.6) years with a male: female ratio of 1:1.5 (Table 1). The online questionnaire results regarding individual perception of essentiality and difficulty of knowledge and skills in RP are presented in Fig. 1A–D. The three most essential topics were denture design, basic components of removable dentures, and occlusion (Fig. 1A). The participants rated occlusion, dental materials, and denture design as the most difficult topics (Fig. 1B). Regarding essential skills, the participants rated impression making, patient communication, and laboratory work evaluation as the most important (Fig. 1C). However, they perceived establishing the vertical dimension, impression making, and bite registration to be the most challenging skills to develop (Fig. 1D). The answers obtained from open-ended questions were used as a guide for creating the questions for focus group interview. The comments were similar to those retrieved through qualitative focus group interviews. Figure 2 depicts the factors associated with knowledge and skill development.

**Table 1:** Characteristics of the participants

Characteristics	% Distribution (by column)		
	Pre-clinical students (*n* = 146)	Clinical students (*n* = 131)	Recent graduates (*n* = 45)
Age (years): mean ± SD	21.6 ± 0.9	23.7 ± 0.8	25.2 ± 1.4
Sex			
Male Female	41.8 58.2	40.5 59.5	40.0 60.0
Educational level			
3rd year student 4th year student 5th year student 6th year student Recent graduates Recent graduates currently pursued higher education	24.0 76.0 - - - -	- - 54.2 45.8 - -	- - - - 73.3 26.7

Qualitative analysis was employed to triangulate the quantitative results. The thematic analysis unveiled 4 threshold concepts (Table 2), encompassing the fundamentals of RP, removable partial denture design, masticatory system and occlusion, and dental materials. The fundamentals of RP included partial denture components, biomechanics of removable dentures, and clinical sequences and workflows. The students learned each topic separately, lacking a clear understanding of their interconnection. This fragments process hindered the application of gained knowledge in clinical practice, leading to reliance on memorization without comprehension.

**Table 2:** Participants’ statements relating to the theme of threshold concept

Threshold concepts		Troublesome	Transformative	Integrative	Liminal	Irreversible
**Fundamental of RPs**	**Biomechanics of removable dentures**	“I’m confused after learning these topics, and I can’t quite grasp the overall picture. I followed the lessons, but I didn’t really understand much.” (Fourth-year V)	N/A	N/A	N/A	N/A
	**Procedural sequences and treatment planning**	“I’ve learned many steps, but I couldn’t connect them together.” (Fourth-year F)			“I understand what I’ve learned, but I can’t adapt it for clinical use. Certain topics, like tissue border molding, are particularly difficult to grasp.” (Fourth-year T)	N/A
		“I don’t understand what needs to be done first or next. It’s as if I’ve learned these topics separately, but I can’t see the whole picture of what will happen.” (Fourth-year V)	N/A	N/A		
	**Border mold & Impression making**	“When studying, I don’t understand at all. I only know that modeling compound was used to make borders, but I don’t know how to actually do it.” (Sixth-year G)	N/A	N/A	N/A	N/A
**RPD Design**		“There isn’t just one correct design. There are always many possible designs, but I don’t know which one is better than the others. I can’t make decisions on my own.” (Fifth-year K)	“The condition in the patient’s mouth is not ideal; there are various details that need adjustments. That’s when I realized how important those lectures are.” (Fifth-year P)	“The subject of design is like the integration of the topics we have learned, including basic components, anatomy, and biomechanics.” (Sixth-year P)	“I understood the indications for those clasps. However, when tasked with designing an RPD for a patient with a different oral condition from what I had practiced, I feel uncertain about how to proceed.” (Fourth-year H)	“After studying for a while, I understand it and don’t have to revise much before designing an RPD.” (Sixth-year M)
		“During the oral examination, we need to consider the design, which components to use, and where to place them. However, at that moment, I couldn’t recall it.” (Fifth-year N)				
**Occlusion**		“I still don’t understand what CR and CO really are, so I just memorize the words and their definitions.” (Fourth-year V)	“Occlusion is an essential aspect present throughout the treatment.” Fifth-year P	“The selection of occlusion for complete dentures is crucial and requires consideration on a case-by-case basis, taking various factors into account.” (Recent graduate J)	N/A	N/A
		“There are some topics that are quite challenging, like the eccentric movement of the jaw. I couldn’t picture it.” (Fourth-year S)				
		“I still don’t know which type of occlusion is more suitable for each class of patients, which tooth should be the guiding tooth, which tooth should be discluded.” (Sixth-year T)				
**Dental materials**		“When I studied these lectures in my second year, I had no idea what these materials were for. I only understood it later.” (Fourth-year K)	N/A	N/A	N/A	N/A
		“I’ve learned the properties of each material but still can’t apply them to clinical use.” (Fifth-year R)				
		“I think it’s difficult because we studied those topics during the second year, and we didn’t know how to apply them for practical use.” (Sixth-year P)				

N/A, not applicable. CO, centric occlusion; CR, centric relation; RPD, removable partial denture

The second challenge was selecting and designing dentures that are appropriate for individual patients. Fourth-year students often experience a liminal phase in which they believe they grasp the various components of denture design, but still struggle to integrate existing knowledge when it comes to denture design. In contrast, fifth-year students begin to merge and apply their prior knowledge to clinical practice. In terms of the masticatory system and occlusion, the students encountered difficulties in envisioning three-dimensional movement, particularly those of the jaw and muscles. To clearly understand occlusion and articulation, the students needed to connect their knowledge with preceding subjects, such as dental and head-and-neck anatomy. Although they comprehended the theory, they lacked confidence in selecting the optimal treatment option for individual patients, such as determining the appropriate occlusal scheme.

Dental materials were another threshold concept for dental students due to its diverse content and technical terms. Similar to the fundamentals of RP, many students feel overwhelmed about whether they can synthesize the overarching concept and connect with the clinical context. Consequently, they encounter challenges in translating theoretical knowledge into practical decision-making, such as selecting the appropriate materials for specific clinical procedures and patient conditions.

Tacit knowledge in RP encompassed skills in border molding and impression making, material handling, and others, such as tooth alteration and recording the maxillomandibular relation at centric relation (Table 3). Impression making posed challenges because the students encountered difficulty in capturing the required anatomical details due to inadequate patient manipulation skills. Handling different materials presented a complexity due to the distinct physical properties of each material, such as consistency and working time. This complexity is particularly pronounced for beginners, especially when making an impression where material control and patient management are simultaneously required. Students also faced challenges in effectively controlling the dental-bur axis to attain an optimal path of insertion, and manipulating the patient’s mandible during centric relation recording.

**Table 3:** Participants’ statements relating to the theme of tacit knowledge

Tacit knowledge	Problems
**Border molding and impression making**	“I couldn’t figure out which angle to insert the tray, how strong to press it, and how to mold the lips and cheeks – I haven’t practiced them together enough. So, when I do the actual impression, it becomes challenging.” (Fifth-year T)
	“The alginate didn’t reach the vestibule in the frontal area when I make the impression and I didn’t know how to do it better.” (Fourth-year S)
	“I can evaluate my work and know whether it is acceptable or not, but the challenge is how to get to that desired outcome.” (Sixth-year G)
**Material handing**	“I couldn’t mix the alginate quickly enough; by the time I tried to put it into the mouth, it had already set.” (Fourth-year F)
	“There are many factors to manage at the same time, even I thought I load enough polysulfide in the tray, once I make the impression, I can see that it’s not. I need to press the tray more firmly.” (Fifth-year P)
	“Managing the modeling compound is extremely difficult; I don’t know how long I need to heat it. Sometimes it takes too long, and it flow everywhere or even burn my hands.” (Fifth-year G)
**Other skills**	“When it comes to tooth alteration for RPD, I know how the bur axis should be, but I never sure that I can control the bur axis correctly and not sure that I can do the same as the trial preparation cast.” (Fifth-year N)
	“It’s quite challenging as we haven’t had any practice in the laboratory class before.” (Sixth-year N)
	“For patient who needs to raise the VD, it’s challenging because I must determine the VD by myself and no reference from their existing occlusion to guide the inter-arch relationship. Similarly, bite registration, it requires the patient’s cooperation to perform the correct or desired bite position (such as CR).” (Sixth-year T)
	“I felt struggled when I manipulate the jaw into CR position, I don’t know that it is patient’s inability to bite at the repeated position or my inability to accurately replicate the CR position.” (Sixth-year P)

**Fig. 3:**
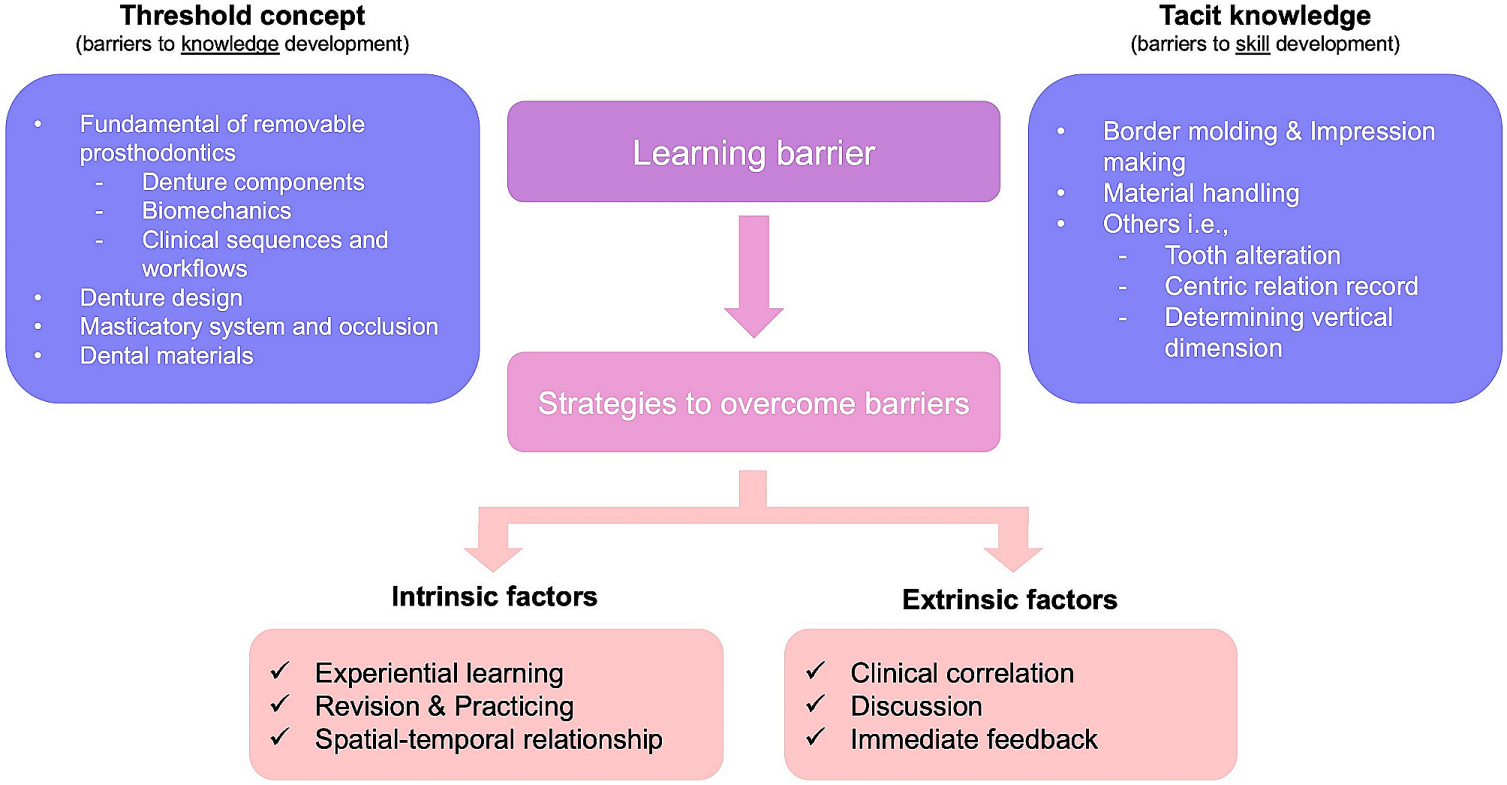
Conceptual framework summarizing the identified themes

The strategies to overcome barriers in achieving RP competencies encompass intrinsic factors, pertained to learners themselves, and extrinsic factors, related to the learning environment (Table 4). Intrinsic factors are divided into self-experience, revision, and spatial-temporal relationship. Engaging with real-world scenarios assisted learners in developing critical thinking and connecting their experiences with existing knowledge and skills. Following initial encounters, they apply these insights to subsequent situations, leading to comprehension and independent execution through review and repetition. The skill of mental imagery, termed spatial-temporal relationship, is critical for understanding intricate topics, such as occlusion. Extrinsic factors include clinical correlations of content, discussions, and immediate feedback. Recognizing the meaningfulness of didactic content by connecting it to real-world applications assists learners in comprehending new knowledge. Observing and engaging in discussions with peers, seniors, or instructors enables experience sharing processes that allow construction and validation of new knowledge. Immediate feedback offers real-time insights into their performance, enabling them to make necessary corrections and improvements. A conceptual framework summarizing the themes derived from the qualitative findings is presented as Fig. 3.

**Table 4:** Participants’ statements on how to overcome learning barriers

Solutions	Intrinsic factors			Extrinsic factors		
	Experiential learning	Revision and practicing	Spatial-temporal relationship	Clinical correlation	Discussion	Immediate feedback
**Fundamental of RP**	“I understand that it there is a certain process with steps to follow, as I’ve done it myself.” (Fifth-year G) “The more I learn about prosthodontics and gain lab experience, the clearer the whole thing gets.” (Recent graduate S)	“Studying it again and revising helped me see the picture more clearly.” (Sixth-year M)	“You have to imagine it as a moving picture in order to understand.” (Sixth-year G)	“Practical knowledge will provide a clearer picture.” (Sixth-year G) “The professor taught about the clinical steps, so I felt that it’s much more relevant.” (Sixth-year T) “When the information is grouped and arranged congruently to the clinical steps, I understand much better.” (Sixth-year P)	“I have to ask seniors or peers from other groups a lot to understand such concepts.” (Fifth-year R) “Observing cases from seniors and instructors is beneficial for learning.” (Recent graduate P)	“The professor tries to help me understand by demonstrations and various explanations when I got confused.” (Recent graduate S)
**RP Design**	“I’ve spent time on my own, and sorting and reflecting my thoughts until I comprehend.” (Sixth-year P) “After undergoing several clinical sections, seeing the patients’ mouth and noticing the differences, I started to understand how I should design the dentures.” (Fifth-year N)	“If I read the lecture more, I’ll understand better.” (Fourth-year H) “When designing, I based on the lecture I’ve read, matching the indications with the appropriate components.” (Fifth-year P)	“People who are good at visualizing can understand and conceptualize designs in their minds.” (Sixth-year M)	“The instructor explains the timeline from the lab to the clinic, outlining the steps one by one. This helps me understand better what needs to be done and why.” (Fourth-year S)	“Active learning allows us to gain knowledge through discussions.” (Sixth-year T) “When new cases differ from what I’ve encountered before, I’ll ask my friends for advice.” (Sixth-year P)	N/A
**Occlusion**	“When I encounter issues in clinic. I have to go back to think and then discuss it with my instructor to understand the problems.” (Sixth-year G)	“I need to listen to the lecture again, repeating. Then, I’ll gradually start to understand better.” (Fourth-year H) “After lecture classes, I have to read what I note again and think thoroughly so that I can understand better.” (Fourth-year V)	“It requires visualizing and time to comprehend these topics.” (Forth-year V)	“It would be great to learn about cases that aren’t ideal but can be frequently found, especially in interesting cases.” (Fifth-year P)	“In the removable partial denture lab, after arranging teeth for occlusion designing, the instructor will guide and provide discussion.” (Sixth-year G)	“I feel like I understand more if I receive feedback after completing the work.” (Sixth-year G)
**Dental materials**	“In laboratory classes, I had the opportunities to try using those materials, like adding more liquid or more powder to see the results, that’s when I understand what I learn in lecture classes.” (Fifth-year P)	“At first, I couldn’t remember anything I’d learnt, but now I understand more after revising over and over again.” (Recent graduate Y)	N/A	“The content should start from the big picture and then move to the smaller points, providing an understanding of the workflow before delving into the details.” (Sixth-year T)	N/A	N/A
**Impression making**	“By getting hands-on experience and encountering real-life practicing, gradually, I am able to make an impression.” (Fifth-year R) “I’ve tried border molding several times, but it didn’t work. However, once I tried thinking and observing closely until I identified the problem, then attempted to fix it, I was able to succeed.” (Fifth-year P)	“The first time might feel difficult, but it becomes easier in subsequent cases.” (Fifth-years G)	“I have to imagine which action will mold which part of vestibule.” (Sixth-year M)	N/A	“The supervisor needs to explain and demonstrate to make it easier to understand.” (Sixth-year P) “Observing what my friends have done and comparing with what I’ve done, and then asking them how they did it.” (Sixth-year G)	“After trying it for the first time, receiving feedback on what needs improvement from others can help us improve.” (Fourth-year H) “The instructor comes and advises where I should apply more pressure, then points out the cause of problems in my first try. Then, the second or third attempt was improved a lot.” (Fourth-year T)
**Material handling**	“I need more experience to determine when to load more material during the impression, compared to the usual amount.” (Fifth-year P)	“I have to practice mixing more than this until I become proficient.” (Fourth-year F) “Practicing until we become proficient in the lab makes it much easier when working in the actual clinic.” (Fifth-year T)	N/A	N/A	N/A	“I’ve made occlusion rims before, and when my supervisor gives feedback for adjustments, I can remember what it should be.” (Sixth-year G)

N/A, not applicable

## Discussion

The present study identified significant barriers that compromise learning in RP in terms of threshold concepts and tacit knowledge. To achieve RP competency, students need to acquire theoretical knowledge and psychomotor skills, then integrate both aspects into clinical practice [5]. Strategies to overcome these obstacles include intrinsic and extrinsic factors with the adoption of experiential learning. In this study, dental students’ comprehension of each topic and the difficulty they encountered were assessed through interviews rather than relying on examination scores or grades. This approach was chosen because previous research has shown that grade point averages and objective tests may not fully reflect the deep learning of individuals [21]. Deep learning encompasses activities such as integrating knowledge, making deeper meaning of the information, and creating interconnected memorization [22].

Mastering the fundamentals of RP and denture design is essential before integrating and applying the knowledge in actual clinical situations for individual patients. These topics become threshold concepts due to their troublesome and integrative nature [8, 9, 11]. Knowledge acquisition in RP requires an understanding of the oral tissue and biomechanics involved in denture components and movement. This understanding is crucial for designing a dental prosthesis that aligns with the patient’s masticatory system. However, students may struggle to connect and integrate basic knowledge to different clinical contexts or individual patients. This may result from lacking of the clinical correlation between the learned content and its applications.

Due to the intricacies of threshold concepts, some students may get stuck within the liminal state, lacking scaffolds or essential frameworks to assimilate new information based on their existing knowledge [7–9]. Becoming stuck in the liminal state could be due to cognitive overload, resulting from an overwhelming amount of information presented within a limited timeframe. This situation aligns with the cognitive load theory, which highlights the limitations of human working memory [23]. Students can potentially progress towards the zone of proximal development, where their clinical performance can flourish with adequate prior knowledge, together with guidance and support from their supervisors or peers before achieving autonomy [24].

Developing clinical skills becomes challenging for novices and beginners because they lack clinical experience. This complexity arises from the elusive nature of tacit knowledge that is not easily conveyed verbally due to its association with psychomotor learning, especially concerning proprioception and hand control [13]. These processes take place within the basal ganglia, a distinct region separate from the frontal lobe where cognitive-based learning occurs [13]. Passive methods, such as reading instructions, verbal explanation, and visual demonstration, are insufficient for effective clinical performance. Instead, learners must internalize sensory feedback to cultivate psychomotor learning. From the present findings, learners often encounterd initial challenges when performing tasks that require new skills. However, most learners gradually acquire psychomotor proficiency through repetitive practice.

Students across various academic years exhibited diverse perspectives and strategies in tackling the problems, influenced by their distinct clinical experience. This phenomenon aligns with the novice-expert continuum [2]. Pre-clinical students commonly report cognitive overload when facing new, intricate topics, struggling with lab tasks, or uncertainty on achieving desired outcomes. This might be due to a transition from novices to advanced beginners, where cognitive models remain unstructured and psychomotor ability is being developed and co-ordinated across different neural pathways. This stage requires extensive trial-and-error efforts over time. Meanwhile, clinical students face the main challenge of translating their acquired knowledge and lab skills to real-life situations. With accumulated experience, they gradually overcome these barriers with subsequent experiences and begin to observe sequential changes. These findings align with the previous literature, demonstrating clinical students’ progression towards a competent stage through integration of knowledge, skills, and attitudes into practical contexts [4]. Recent dental school graduates demonstrate their competence by self-assessing their abilities to determine suitable tasks and provide appropriate treatment. Competent practitioners possess a clear awareness of their competency level, which allows them to determine whether they can manage or should refer patients to proficient or expert practitioners [2, 4].

Based on our findings, the proposed strategies for overcoming threshold and tacit knowledge in RP learning encompass internal and external factors. Internal factors include self-experience, revision, and spatial-temporal relationships. Self-experience linked to experiential learning assists learners in forming concepts through their actions and mistakes, promoting deep understanding and performance. This approach enables learners to solidify their own understanding and assess its alignment with reality before clinical application [13]. Consistent knowledge and skill development are fostered though revisions as a part of repetitive learning, which comprises memorizing facts, revision, repetitive practice, critical reflection, and constructive feedback from instructors [15]. Spatial-temporal relationships enable the visualization of complex object relationships [17], particularly in topics requiring visualizing movement or mental imagery, such as the functional movement of removable partial dentures or mandibular movement. Students with limited spatial perception may face challenges, however, active learning and visualization can mitigate this as most students could establish abstract conceptualizations despite a lack of clinical exposure or practical experience. Instructors can assist learners through quizzes or exercises that encourage the application of prior knowledge and provide regular opportunities for practical application in laboratories or clinical settings, thereby completing the experiential learning cycle.

External factors encompass learners’ exposures within the learning process, including clinical correlations, discussions, and immediate feedback. Clinical correlations enhance learning significance by linking new information to existing knowledge [9], while discussions enable collaborative knowledge construction through shared experiences and communication [16]. Immediate feedback from supervisors offers real-time learners’ performance insights, assisting in correction and skill improvement, essential for developing proprioception, hand position, and movement sense [13], while preventing undesirable habits or muscle memory [10].

Our findings demonstrated an important role of instructors in effective teaching by rendering knowledge meaningful. This involves demonstrating congruency between pre-clinical knowledge and clinical scenarios for pre-clinical students, and linking current clinical encounters with pre-clinical knowledge for clinical students. Moreover, the learning environment should facilitate opportunities for discussions, which can be achieved by employing active learning methods that encourage knowledge exchange among individuals. For skill development, instructors should attentively observe learners’ performance, address mistakes, and provide immediate corrective demonstration [13]. These strategies enhance deep knowledge acquisition and vital skill honing, overcoming barriers in learning RP and benefiting academic staff in refining RP teaching.

The present study has some limitations. The questionnaire’s response rate fell below the expected value. This low response rate could introduce non-response bias. It is conceivable that dental students and recent graduates with unfavorable attitudes towards RP, including those who lacked comprehension of the RP concept or no longer engaged in clinical practice related to removable prosthesis fabrication, might be unwilling to respond to the questionnaire. However, it is not meaningful because the questionnaire served as a tool for creating questions for focus group interviews. Qualitative research can introduce bias and subjectivity due to the investigators’ interpretation [25]. To mitigate this, reflexivity (experiences, beliefs, or assumptions of the investigators) is applied to explain personal thoughts more objectively [26]. This study separated neutral sections (literature review, methodology, and demographic information) from value-influenced parts (data summarizing, analysis, and discussion). Conducted in a single dental school, the transferability of the findings may be limited due to the different characteristics of students and instructors as well as the learning environment from other dental schools. Although variations in dentistry fields might affect the applicability of our results, this study’s approach can serve as a framework for exploring learning barriers in other contexts. Further studies on a larger population with different backgrounds and other dentistry fields are recommended.

## Conclusion

From the present findings, the threshold concept for achieving RP competency includes RP fundamentals, partial denture design, masticatory system, occlusion, and dental materials. Two key tacit skills for achieving RP competency are impression making and material handling. Effective teaching and learning strategies to address these challenges involve intrinsic and extrinsic factors, with the adoption of experiential learning. Our findings suggest effective learning strategies and teaching methods to maximize student learning and competency development when designing the undergraduate RP curriculum in dental education.

### Electronic supplementary material

Below is the link to the electronic supplementary material.

Supplementary file 1: Questionnaire for Removable Prosthodontics

## Data Availability

Dataset generated during the current study is available upon request to the corresponding authors.
