# Supplementary material for: Identifying barriers and strategies for achieving competency in removable prosthodontics in undergraduate dental students: a mixed-method study
Source: BMC Oral Health. 2024 Jan 23;24:123. doi: 10.1186/s12903-024-03874-x (PMC10804482; doi:10.1186/s12903-024-03874-x)
Supplement: Supplementary file 1 — Questionnaire for Removable Prosthodontics [file 12903_2024_3874_MOESM1_ESM.pdf]

Questionnaire for Removable Prosthodontics

Part I: Personal information

Sex: ❒ Male ❒ Female ❒ Not indicated

Current year of education:

❒ 3rd year ❒ 4th year ❒ 5th year ❒ 6th year

❒ recent graduates (academic year 2021 or 2022), not yet pursuing higher education

❒ recent graduates (academic year 2021 or 2022), pursuing higher education

Part II. Closed-ended questions

Section II.1) Lecture & Laboratory learning (for all participants)

1.1) Please indicate your level of understanding of the content in the **Complete Denture lectures**.

Note: The evaluation should be based on overview perspective regardless of your grade

❒ Very good ❒ Good ❒ Fair ❒ Poor

1.2) Please indicate your level of understanding of the contents in the **Removable Partial Denture lectures**.

Note: The evaluation should be based on overview perspective regardless of your grade

❒ Very good ❒ Good ❒ Fair ❒ Poor

1.3) Please indicate your level of understanding of the concepts and developing hand skills related to **Complete Denture laboratory**.

Note: The evaluation should be based on overview perspective regardless of your grade

❒ Very good ❒ Good ❒ Fair ❒ Poor

1.4) Please indicate your level of understanding the concept and developing hand skills related to **Removable Partial Denture laboratory**.

Note: The evaluation should be based on overview perspective regardless of your grade

❒ Very good ❒ Good ❒ Fair ❒ Poor

Section II.2) Theoretical learning

2.1) Please indicate the level of essentiality of the following topics and knowledge for achieving success in removable prosthodontics (only for the 5th and 6th year students & Recent graduate)

	Very Essential	Essential	Unessential	Very unessential
1. Dental anatomy				
2. Oral anatomy				
3. Oral histology				
4. Occlusion				
5. Dental materials				
6. CD & RPD theory				
7. CD & RPD design				
8. Fixed prosthodontics				
9. Oral examination principle				
10. Oral surgery				
11. Operative dentistry				
12. Endodontics				
13. Oral pathology				
14. Radiology				
15. Community dentistry				
16. Oral epidemiology				
17. Infection control				
18. Patient evaluation				

Note: CD, complete denture; RPD, removable partial denture

2.2) Please indicate the level of difficulty in learning and understanding of the following topics in general. (for all participants)

Operational definition

Very easy to understand = be able to understand after a classroom learning or self-reading without requiring instructors' explanation

Easy to understand = be able to understand after revising or receiving additional explanations from friends, instructors, or others

Difficult to understand = requiring repeated explanations from friends, instructors, or others before understanding

Very difficult to understand = be unable to understand even up to the present

	Very easy	Easy	Difficult	Very difficult
1. Dental anatomy				
2. Oral anatomy				
3. Oral histology				
4. Occlusion				
5. Dental materials				
6. CD & RPD theory				
7. CD & RPD design				
8. Fixed prosthodontics				
9. Oral examination principle				
10. Oral surgery				
11. Operative dentistry				
12. Endodontics				
13. Oral pathology				
14. Radiology				
15. Community dentistry				
16. Oral epidemiology				
17. Infection control				
18. Patient evaluation				

Note: CD, complete denture; RPD, removable partial denture

2.3) Please rank the three most crucial factors in fostering knowledge and understanding when studying theoretical subjects. (for all participants)

❒ Learning sequence

❒ Teaching materials/media

❒ Instructors’ ability to articulate and clarify

❒ Opportunities for learners to inquire

❒ Self-revision

❒ Doing exercises

❒ Observing the actual work situation (ได้เห็นภาพการทำงานจากสถานการณ์จริง (เจอในแล็บ/คลินิก))

❒ Others……………………….

Section II.3) Clinical practice learning

3.1) Please indicate the essentiality of the following skills for achieving success in removable prosthodontics (only for the 5th and 6th year students & Recent graduate)

	Very Essential	Essential	Unessential	Very unessential
1. Oral examination				
2. Dental bur use (i.e., for occlusal adjustment, tooth alteration)				
3. Making preliminary impression				
4. Making final impression				
5. Facebow use				
6. Dental prosthesis design				
7. Determining vertical dimension				
8. Determining centric relation/centric occlusion				
9. Study model fabrication				
10. Mounting cast to the articulator				
11. Artificial teeth arrangement				
12. Clasp bending				
13. Baseplate and occlusion rim fabrication				
14. Evaluating laboratory work				
15. Dentist-patient communication				
16. Dentist-lab technician communication				
17. Working in an ergonomic posture				

3.2) Please indicate the difficulty in performing clinical practice regarding the following steps/procedures (only for the 5th and 6th year students & Recent graduate)

Operational definition

Very easy to perform = be able to complete the task independently or with minimal guidance

Easy to perform = be able to accomplish the task largely independently, yet occasionally requiring some assistance from friends, instructors, or others

Difficult to perform = be able to accomplish the task somewhat independently, but requiring significant assistance from friends, instructors, or others to complete the task

Very difficult to perform = requiring ongoing assistance, and not yet achieve the task independently up to the present

	Very easy	Easy	Difficult	Very difficult
1. Oral examination				
2. Dental bur use (i.e., for occlusal adjustment, tooth alteration)				
3. Making preliminary impression				
4. Making final impression				
5. Facebow transfer				
6. Dental prosthesis design				
7. Determining vertical dimension				
8. Determining centric relation/centric occlusion				
9. Study model fabrication				
10. Mounting cast to the articulator				
11. Artificial teeth arrangement				
12. Clasp bending				
13. Baseplate and occlusion rim fabrication				
14. Evaluating laboratory work				
15. Dentist-patient communication				
16. Dentist-lab technician communication				
17. Working in an ergonomic posture				

3.3) Please rank the three most crucial factors in building and developing practical learning skills

(only for the 5th and 6th year students & Recent graduate)

❒ Teaching materials/media

❒ Instructors' ability to articulate and clarify

❒ Instructors' feedback and suggestions during practicing

❒ Self-repetitive practice

❒ Doing exercises

❒ Working and practicing in the actual work situation

❒ Observing laboratory and clinical demonstration

❒ Others……………………….

Part III. Open-ended question (for all participants)

Please provide additional comments on the difficulty and necessity of each topic regarding knowledge and skill development for successful removable prosthodontic treatment.

…….

…….

…….
